# Carbon dynamics and environmental controls of a hilly tea plantation in Southeast China

**DOI:** 10.1002/ece3.5504

**Published:** 2019-07-31

**Authors:** Jiaping Pang, Hengpeng Li, Xuguang Tang, Jianwei Geng

**Affiliations:** ^1^ Key Laboratory of Watershed Geographic Sciences, Nanjing Institute of Geography and Limnology Chinese Academy of Sciences Nanjing China; ^2^ Chongqing Key Laboratory of Karst Environment, School of Geographical Sciences Southwest University Chongqing China; ^3^ University of Chinese Academy of Sciences Beijing China

**Keywords:** carbon dioxide, eddy covariance, net ecosystem CO_2_ exchange, tea plantation

## Abstract

Tea plantations are widely distributed and continuously expanding across subtropical China in recent years. However, carbon flux exchanges from tea plantation ecosystems are poorly understood at the ecosystem level. In this study, we use the eddy covariance technique to quantify the magnitude and temporal variations of the net ecosystem exchange (NEE) in tea plantation in Southeast China over four years (2014–2017). The result showed that the tea plantation was a net carbon sink, with an annual NEE that ranged from −182.40 to −301.51 g C/m^2^, which was a much lower carbon sequestration potential than other ecosystems in subtropical China. Photosynthetic photon flux density (PPFD) explained the highest proportion of the variation in NEE and gross primary productivity (GPP) (for NEE: *F* = 389.89, *p* < .01; for GPP: *F* = 1,018.04, *p* < .01), and air temperature (T_a_) explained the highest proportion of the variation in ecosystem respiration (RE) (*F* = 13,141.81, *p* < .01). The strong pruning activity in April not only reduced the carbon absorption capacity but also provided many plant residues for respiration, which switched the tea plantation to a carbon source from April to June. Suppression of NEE at higher air temperatures was due to the decrease in GPP more than the decrease in RE, which indicated that future global warming may transform this subtropical tea plantation from a carbon sink to carbon source.

## 1 INTRODUCTION

Tea plantations are widely cultivated in China, with a total area that approached 3.0 × 10^6^ ha in 2016, which was mostly distributed in the subtropical regions of China (NBSC, 2017). In recent years, tea plantations have expanded continuously in hilly areas of Southeast China, and many forests have been cleared and converted to tea plantations. The dramatic increase in tea plantations and strong plantation management practices (pruning and fertilization) have resulted in a series of environmental issues, such as acidification of the soil (Guo et al., 2010; Li et al., 2016; Yang et al., 2018), nitrate leaching into the surrounding water systems (Liu, Yang, Yang, & Zou, 2012; Xu et al., 2017), and high rates of N_2_O emissions to the atmosphere (Chen et al., 2017; Fu et al., 2012, 2015; Yao, Wei, Liu, Zheng, & Xie, 2015). This land use change may also affect regional carbon budgets (Ingrisch et al., 2018; Thom, Rammer, Garstenauer, & Seidl, 2018), yet the understanding of the carbon dynamics in tea plantation is still poor (Chiti, Diaz‐Pines, Butterbach‐Bahl, Marzaioli, & Valentini, 2018; Kamau, Spiertz, & Oenema, 2008; Li et al., 2011). The study of carbon exchange and its responses to the management practices and environmental variables of tea plantation ecosystems is crucial to improve our understanding of the carbon fixation process and the drivers in subtropical tea plantations.

Many studies have reported that the variation of NEE depends on the response of its components’ GPP and RE to changes in environmental conditions (Ahlstrom et al., 2015; Baldocchi, 2008; Reichstein et al., 2013; Yu et al., 2008). When soil water is not limited, GPP is mainly controlled by the PPFD, and RE is controlled by soil temperature (T_s_) (Baldocchi, 2008; Hinko‐Najera et al., 2017; Xie et al., 2015; Yu et al., 2008). In addition, GPP and RE have also been reported as being affected by radiation, T_a_, vapor pressure deficit (VPD), leaf area index (LAI), and soil water content (SWC) (Song, Chen, Zhou, Jiang, & Peng, 2017; Yu et al., 2008; Zhang et al., 2018; Zhu et al., 2015). The responses of GPP and RE to environmental factors in different ecosystems are complex. Some studies have shown that GPP was more sensitive to higher temperatures than RE (Garcia et al., 2017; Wagle, Kakani, & Huhnke, 2015), while other research reported that high temperatures increased RE more than GPP (van Dijk, Dolman, & Schulze, 2005). Furthermore, the widely applied anthropogenic management of strong pruning after tea harvest in a tea plantation also affected the carbon sequestration process of the plantation (Li et al., 2011), but the impact of this management on NEE and its environmental drivers has not yet been evaluated.

In the last few decades, the eddy covariance (EC) technology has been increasingly used as direct CO_2_ and water vapor flux measurements between the vegetation and the atmosphere at the ecosystem scale (Baldocchi, 2003, 2014). These continuous and uninterrupted flux data are essential for improving our understanding of carbon dynamics and their interactions with different environmental factors at different temporal and spatial scales. East Asia's monsoon subtropical forests have been demonstrated to have an important and high carbon dioxide uptake (Tang et al., 2012; Yu et al., 2014), yet most of the carbon budget studies in this region have focused on natural ecosystems, such as bamboo forests (Chen, Jiang, Cai, Zhou, & Peng, 2018; Song et al., 2017), coniferous plantations (Sun, Wen, Yu, Liu, & Liu, 2006; Yu et al., 2005), mangrove wetlands (Cui et al., 2018), and *Populus dettoides* plantations (Gao et al., 2015). The study of carbon dynamics of the ongoing expansion of tea plantations and the impact of pruning management allows us to better understand the regional carbon budgets in subtropical regions. This study should also provide theoretical guidance for regional carbon management techniques to mitigate climate change.

Here, an eddy covariance technique was used to measure the seasonality of the CO_2_ flux above a tea plantation in Tianmuhu watershed, Southeast China from 2014 to 2017. The main objectives of this study were (a) to quantify the magnitude and temporal variations (diurnal, seasonal, and annual) of NEE and its components, GPP and RE, using the eddy covariance method in a tea plantation; and (b) to identify the effect of environmental variables and management on CO_2_ flux variations.

## 2 MATERIALS AND METHODS

### 2.1 Site description

The tea plantation study site for CO_2_ and water vapor flux measurements belongs to the field plot of the Key Laboratory of Watershed Geographic Sciences, Nanjing Institute of Geography and Limnology, Chinese Academy of Sciences (31°16′14ʺN, 119°27′15ʺE). This site is in the upstream area of the Taihu region and is characterized by a subtropical monsoon climate with warm‐humid summers and cold‐dry winters. The mean annual precipitation is 1,155.8 mm, and the mean annual air temperature is 15.8°C. The precipitation has an uneven temporal distribution, with 76% of the annual precipitation distributed between March and September. The local soil is a combination of Lateritic red soil and yellow soil, which is characterized by a high gravel content (~25%) and shallow profile (<0.6 m). This tea plantation was transplanted in 2010 on an area of approximately 30 ha, with a plant density of 41,250 plants/ha and an inter‐row spacing of 1.5 m. The canopy height is approximately 0.7 m. The management practices include fertilization and pruning activities.

### 2.2 Instrumentation and data acquisition

The EC flux tower was established in the center of tea plantation in January 2014 to continuously measure CO_2_ and water vapor fluxes. The EC instruments were installed at a 3 m height, with a three dimensional sonic anemometer (CSAT‐3, Campbell Scientific) to measure the three components of wind velocity vectors and sonic temperature and an open‐path infrared gas analyzer (EC150, Campbell Scientific) for acquiring the CO_2_ and water vapor air concentrations. The raw data were sampled at 10 Hz, and the 30 min mean fluxes were logged by a Campbell Scientific CR3000 data logger (CR3000, Campbell Scientific).

Simultaneously, a suite of meteorological and environmental data was also measured by meteorological instruments mounted on the flux tower. Air temperature and relative humility (Model HMP 155A, Campbell Scientific) as well as the wind speed (Model 010C, Met One Instruments Inc.) were measured at the same height of the EC instruments. A four‐component net radiometer (Model CNR‐4, Kipp & Zonen Inc.), a quantum sensor of photosynthetically active radiation (Model PQS1, Kipp & Zonen Inc.), and a pyranometer (Model CMP3, Kipp & Zonen Inc.) were mounted at ~3 m and used to measure radiation. The vertical profiles of soil temperature and soil water content were measured with three soil probes (Model CS 650‐L, Campbell Scientific) installed at 10, 20, and 50 cm depths, respectively. Soil heat flux was measured using two plates (Model HFP01, Hukseflux) placed at a 5 cm depth. Precipitation was measured with a tipping‐bucket gauge (Model TB4MM, Hydrological Services Pty. Ltd.) mounted at the top of the tower. Rainfall was measured at 30‐min intervals, and all the other environmental variables were measured at 1 Hz, averaged to 30 min, and stored by a data logger (CR3000, Campbell Scientific). All the sensors were powered by solar panels.

### 2.3 Flux data processing

A planar fit rotation of the coordinate was applied to avoid the effects of instrument tilt or irregularity on the airflow (Wilczak, Oncley, & Stage, 2001). The Webb–Pearman–Leuning (WPL) correction was made for density fluctuations resulting from heat and water vapor transfers (Webb, Pearman, & Leuning, 1980).

Spurious data caused by rainfall, water condensation, or system failures were screened and eliminated from the flux dataset (Yu et al., 2006). Flux data under low turbulence conditions (mostly during nighttime) were identified according to the friction velocity (u*) threshold method (Reichstein et al., 2005), while data lower than the u* threshold were excluded to avoid a possible underestimation of the ecosystem respiration. Data gaps occurred due to rainfall, water condensation, system failures, and nighttime u* filtering. The amounts of available data were 43.05%, 32.89%, 54.21%, and 51.56% in 2014, 2015, 2016, and 2017, respectively. From these values, the average amounts of daytime and nighttime valid data were 70.68% and 30.28% (Table 1).

**Table 1:** Data statistics after quality control procedures

Year		Unavailable data (%)^a^	Filtered data (%)^a^	Valid data (%)
2014	Daytime	1.28	39.82	58.90
	Nighttime	1.11	65.34	33.54
	Total	1.18	55.77	43.05
2015	Daytime	26.68	18.11	55.21
	Nighttime	26.81	53.69	19.50
	Total	26.76	40.35	32.89
2016	Daytime	3.57	14.68	81.75
	Nighttime	4.37	57.95	37.68
	Total	4.07	41.72	54.21
2017	Daytime	0.38	12.75	86.86
	Nighttime	1.62	68.00	30.38
	Total	1.15	47.28	51.56

a Unavailable data include data gaps due to instrumental failure, power failure, instrument maintenance, and calibration. Filtered data include data lost due to data filtering procedures.

Data gaps from carbon fluxes were filled based on the gap‐filling procedure of Falge (Falge et al., 2001). NEE partitioning into GPP and ER followed the flux partitioning methods of Reichstein (Reichstein et al., 2005). Additional data quality control, gap‐filling, and flux partitioning procedures are described in detail in Yu et al. (2006). After performing gap filling, the 30‐min flux data were summed to obtain flux budgets at daily, monthly, and annual scales. Positive values of NEE indicate a net carbon source, while negative values represent a net carbon sink. Meteorological data gaps of <2 hr were filled using linear interpolation from adjacent data points. Larger gaps were filled based on the nonlinear regression method.

### 2.4 Energy balance closure

The energy balance closure was used to assess the quality of the eddy covariance measurements. We analyzed the energy balance closure from 30‐min mean available values of latent heat (LE), sensible heat (H), net radiation (R_n_), and soil heat flux (G) for each year as follows:

(1) $$\text{EBR}=\left(\text{LE}+H\right)/\left(R_{n}-G\right)$$

**Figure 1:**
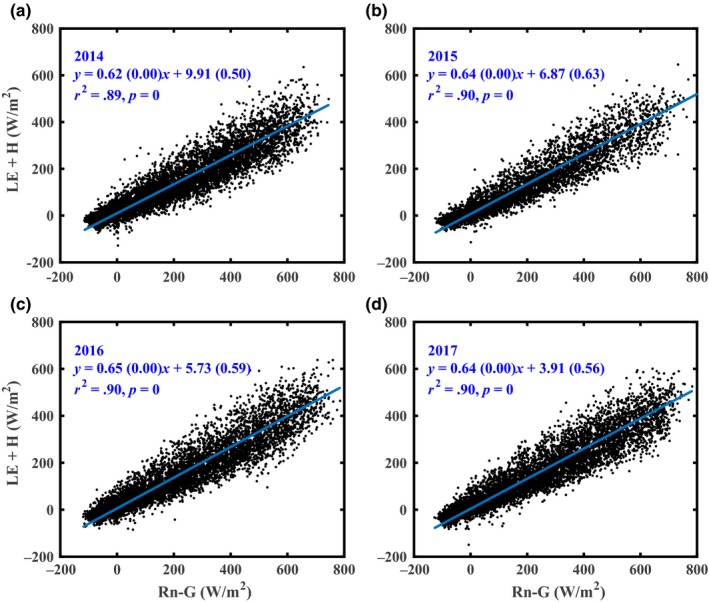
The 30‐min averages of the balance of net radiation (R_n_) minus soil heat (*G*) fluxes plotted versus corresponding values of the sum of latent heat (LE) and sensible heat (H) fluxes over the four years of period (2014–2017). The solid line represents the regression through the points

The energy balance closures were 0.62, 0.64, 0.65, and 0.64 at this tea plantation from 2014 to 2017, respectively (Figure 1). These data are within the range of 0.34 to 1.69 found at other FLUXNET sites (Wilson et al., 2002).

## 3 RESULTS AND DISCUSSION

### 3.1 Meteorological conditions during the study period

**Figure 2:**
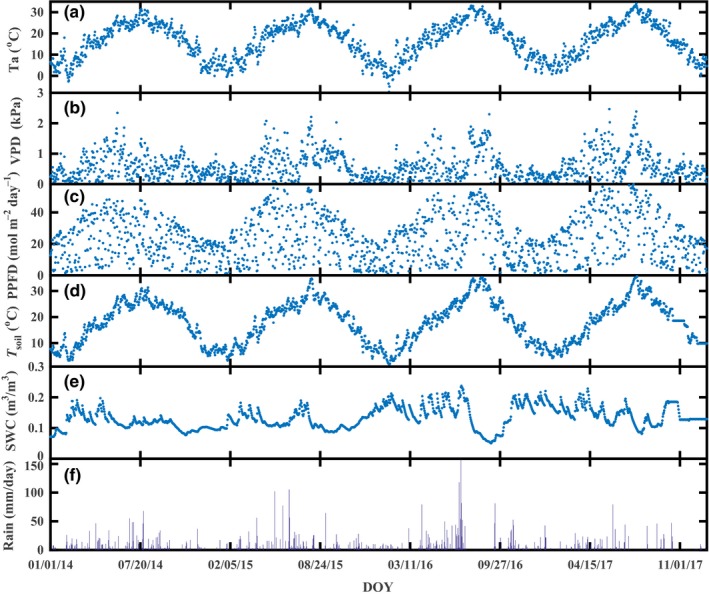
Time series of daily air temperature (T_a_), vapor pressure deficit (VPD), photosynthetic photon flux density (PPFD), soil temperature at 10 cm depth (T_s_), soil volumetric water content at 10 cm depth (SWC), and rainfall measured at the tea plantation in 2014–2017

Seasonal patterns of meteorological conditions at a daily scale for the four studied years are shown in Figure 2. During the investigated period, the T_a_, VPD, PPFD, and T_s_ showed a strong seasonality. The mean daily T_a_ during the entire study period ranged from −7.53 to 34.05°C, with annual mean T_a_ of 16.35, 16.28, 16.81, and 16.85°C for 2014–2017, respectively. Similarly, VPD showed a higher value during summer and lower values during winter, as daily maximum values of 2.34, 2.21, 2.30, and 2.47 kPa were observed for 2014–2017, respectively. PPFD ranged between 0.90 and 57.93 mol m^−2^ day^−1^. T_s_ showed similar seasonal dynamics to T_a_, as the mean annual values were 17.53, 17.63, 18.09, and 18.42°C for 2014–2017, respectively. SWC variation at the depth of a 10 cm was caused by the uneven distribution of precipitation, with mean annual values of 0.12, 0.12, 0.15, and 0.15 m^3 ^m^−3^ observed in 2014–2017, respectively. Precipitation (P) was generally concentrated between April and July, and relatively less rain fell in November to March. An extremely large amount of precipitation was observed in 2016, as the total precipitation was 1,242 mm from April to July, and the maximum daily precipitation was 158.6 mm (2 July, 2016).

### 3.2 Diurnal variations of CO_2_ fluxes

**Figure 3:**
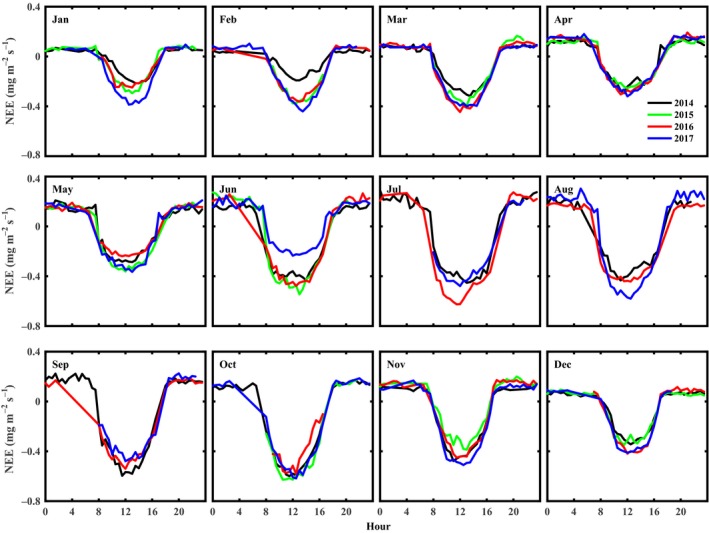
Monthly average diurnal variation of half‐hourly measurements of NEE at the tea plantation in 2014–2017. Monthly averages were calculated from all available measured half‐hourly data

The monthly average diurnal variations of NEE from January to December over the four years of study period are shown in Figure 3. The diurnal variation of NEE followed an obvious sinusoidal dynamic in each month in 2014–2017. The NEE values decreased after dawn and reached a minimum at noon due to the tea plantation absorbing CO_2_ from the atmosphere, while a positive NEE at night indicated the release of CO_2_ from the tea plantation into the atmosphere. This diurnal trend varied substantially in magnitude across different months. The maximum amount of CO_2_ absorbed during the daytime ranged from −0.60 to −0.19 mg m^−2^ s^−1^ in 2014, −0.63 to −0.25 mg m^−2^ s^−1^ in 2015, −0.63 to −0.24 mg m^−2^ s^−1^ in 2016, and −0.62 to −0.24 mg m^−2^ s^−1^ in 2017. The peak diurnal value of NEE increased gradually from January and was largest in the summer months (July and August) and decreased during the winter and spring months. A notable reduction of the peak diurnal value of NEE from April to June was probably caused by pruning activity during April.

### 3.3 Seasonal and inter annual dynamic of CO_2_ fluxes

**Figure 4:**
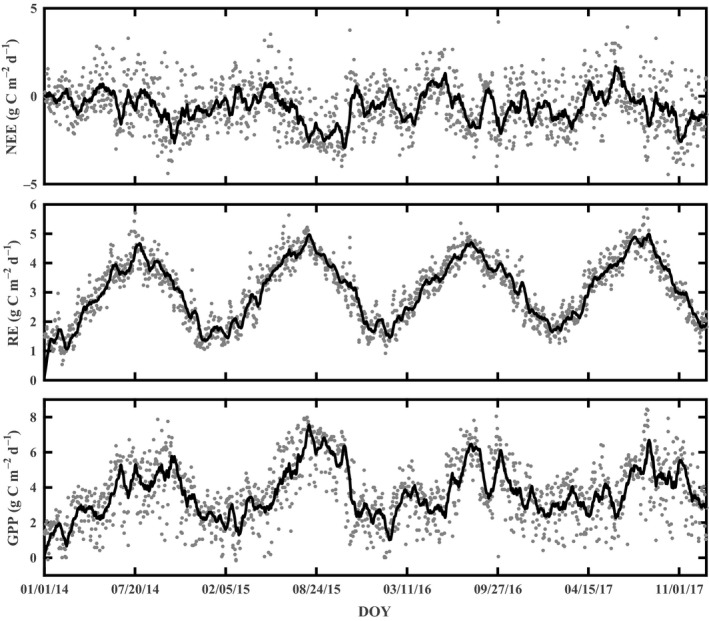
Time series of daily NEE, RE, and GPP measured at the tea plantation from 2014 to 2017. Solid lines for each plot represent the data smoothed with 7‐day moving averages

Daily average NEE, GPP, and RE values showed distinct seasonal patterns and were consistent over four years (Figure 4). The amplitude of daily NEE ranged from −4.39 g C m^−2 ^day^−1^ (on 1 October) to 3.28 g C m^−2 ^day^−1^ (on 5 July), −3.96 g C m^−2 ^day^−1^ (on 19 October) to 3.75 g C m^−2 ^day^−1^ (on 7 November), −4.00 g C m^−2 ^day^−1^ (on 25 September) to 4.22 g C m^−2 ^day^−1^ (on 28 September), and −4.45 g C m^−2 ^day^−1^ (on 7 October) to 3.94 g C m^−2 ^day^−1^ (on 10 July) in 2014–2017, respectively.

RE showed a strong seasonal dynamic throughout the year; during the first two months each year, the RE value was close to 1.5 g C m^−2 ^day^−1^, and after that it rapidly increased and reached its peak value during July; then, it decreased gently to the end of the year. There is a consistent seasonal trend from 2014 to 2017, as the increase and decrease in the respiration component mainly reflected the seasonal dynamic of air temperature.

**Figure 5:**
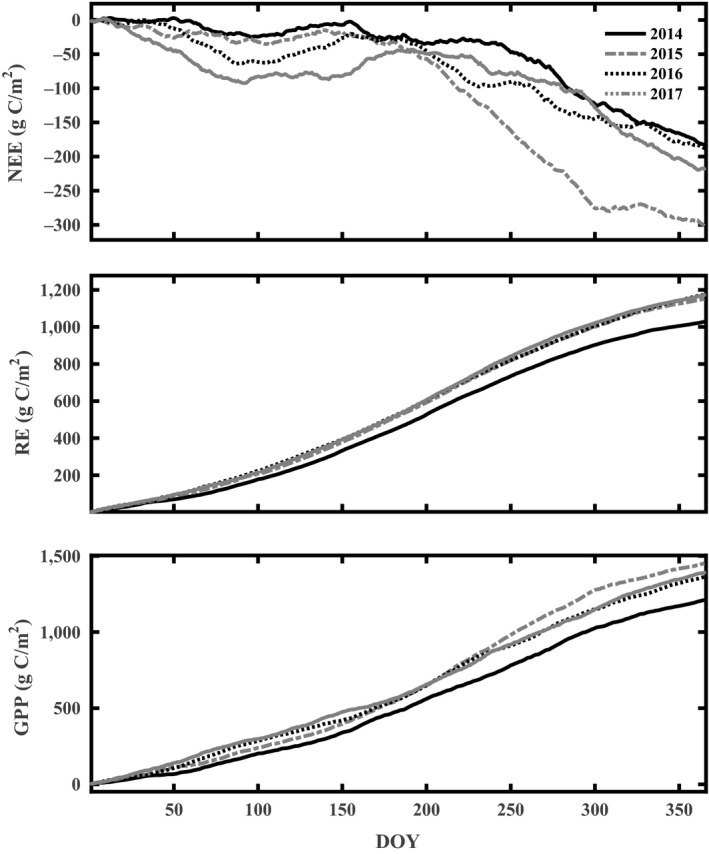
Cumulative fluxes of NEE, RE, and GPP over the experimental period (2014–2017) for the tea plantation

During the first three months each year, GPP values were close to 2  g C m^−2 ^day^−1^, and slowly increased after pruning; the maximum daily GPP values were 7.87 g C m^−2 ^day^−1^ (on 7 September), 7.99 g C m^−2 ^day^−1^ (on 3 August), 8.06 g C m^−2 ^day^−1^ (on 25 September), and 8.47 g C m^−2 ^day^−1^ (on 21 August) in 2014–2017, respectively. Higher photosynthetic active radiation and temperature during the growing season led to a higher GPP flux. During late September, the GPP rate decreased due to senescence of tea plants. The yearly cumulative NEEs were −182.40, −301.51, −189.02, and −218.12 g C/m^2^ in 2014–2017, respectively (Figure 5), which indicated that this tea plantation ecosystem was a carbon sink on an annual scale.

### 3.4 Response of NEE to environmental factors

A multiple stepwise regression analysis was conducted to identify the top three best fitted equations between the daily carbon flux and environmental variables (Table 2). Daily PPFD, T_a_, and P were the most important drivers of NEE, while daily PPFD, T_s_, and P were the most important drivers of GPP. The PPFD explained the highest proportion of the variation in NEE and GPP (for NEE: *F* = 389.89, *p* < .01; for GPP: *F* = 1,018.04, *p* < .01). The daily RE may be driven by T_a_, VWC, and T_s_, and daily T_a_ explained the highest proportion of the variation in RE (*F* = 13,141.81, *p* < .01).

**Table 2:** The multiple stepwise regression analysis of the daily carbon flux (NEE, GPP, and RE) and environmental variables (PPFD, T_a_, P, T_s_, VPD, and SWC) of the tea plantation, 2014–2017

Stepwise regression equation	*F*	*p*	PPFD	T_a_	P	T_s_	VPD	VWC
NEE = 0.480 – 0.049PPFD	389.89	<.01	−0.48					
NEE = 0.01 – 0.065PPFD + 0.051T_a_	279.04	<.01	−0.55	.3				
NEE = −0.084–0.061PPFD + 0.047T_a_ + 0.005P	206.12	<.01	−0.52	.28	.18			
RE = 1.127 + 0.119T_a_	13,141.88	<.01		.95				
RE = 0.692 + 0.122T_a_ + 2.775VWC	7,319.56	<.01		.96				0.3
RE = 0.535 + 0.095T_a_ + 3.234VWC + 0.030T_s_	5,062.24	<.01		.54		0.18		0.35
GPP = −1.830 – 0.084PPFD	1,018.04	<.01	−0.67					
GPP = −0.905 – 0.062PPFD−0.079T_s_	759.14	<.01	−0.53			−0.42		
GPP = −0.975 – 0.058PPFD − 0.083T_s_ + 0.005P	535.77	<.01	−0.5		.18	−0.44		

Note

Partial correlation coefficients between the carbon flux and environmental variables are represented in the PPFD, T_a_, P, T_s_, VPD, and SWC columns.

The relationship between daytime NEE and PPFD (light response curve) was determined each month from April to September across four years by the rectangular hyperbolic function (Michaelis–Menten equation) as follows:

(2) $$\text{NEE}=\frac{\alpha \times \text{PPFD}\times \beta }{\alpha \times \text{PPFD}+\beta }+\text{RE}$$

where *α* is the apparent quantum efficiency, *β* is the carbon uptake capacity at light saturation, PPFD is the photosynthetic active radiation, and RE is ecosystem respiration (see Falge et al. (2001) for details).

**Figure 6:**
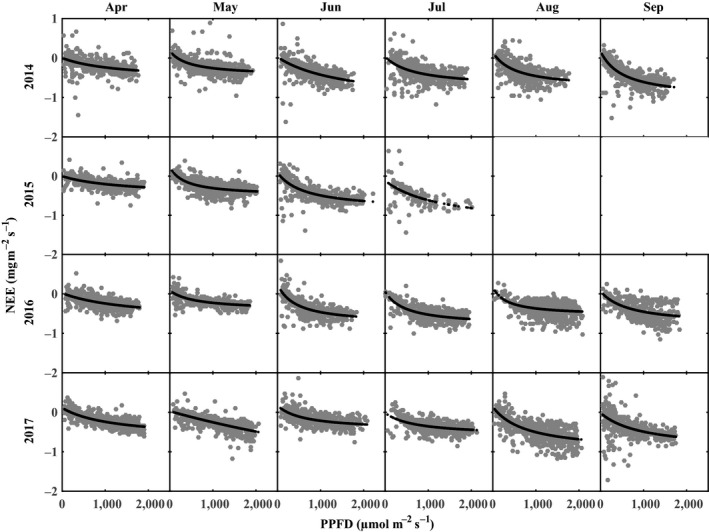
Relationship between day time NEE and PPFD for the tea plantation during April–September over the four years of periods (2014–2017). Half‐hourly data of NEE and PPFD were used to fit the light response curve

The fitted light response curve successfully described the relationship between daytime NEE and PPFD during the growing season (Figure 6). The PPFD explained 12%–49% of the temporal dynamic in daytime NEE for the tea plantation in 2014–2017. The dependence of daytime NEE on PPFD varied among different months of the growing season; this result is due to the changes in plant growth and environmental conditions. The peak value of *α* ranged from 0.0003 to 0.0023, and the corresponding NEE_max_ ranged from 1.0074 to 1.1739 in 2014–2017 (Table 3), which could have been caused by higher temperatures and larger PPFD during the summer. Increased canopy cover during the growing season also leads to increased carbon sequestration potential. The lower values of *α* and NEE_max_ during the early growing season could be caused by a small canopy size after pruning and suboptimal environmental conditions.

**Table 3:** Parameters of the light response curve using a Michaelis–Menten model

Year	Month	*a*	NEE_max_	RE	*R* ^2^	*N*
2014	April	.0004	0.5542	.0013	.20	471
	May	.0014	0.6281	.1790	.28	522
	June	.0007	1.1659	.0244	.37	487
	July	.0011	0.7722	.0338	.24	502
	August	.0018	0.9168	.1449	.38	483
	September	.0023	1.1874	.1708	.57	475
2015	April	.0003	0.4906	.0020	.26	475
	May	.0019	0.7284	.2232	.35	474
	June	.0015	0.9556	.0877	.48	413
	July	.0009	1.1739	.1179	.27	124
	August	–	–	–	–	–
	September	–	–	–	–	–
2016	April	.0004	0.6893	.0182	.32	443
	May	.0008	0.4943	.0836	.44	418
	June	.0023	1.0074	.2394	.49	418
	July	.0018	0.8592	.0533	.46	478
	August	.0022	0.7148	.1689	.16	552
	September	.0013	0.8629	.0671	.32	125
2017	April	.0006	0.7724	.1106	.53	498
	May	–	–	–	–	–
	June	.0010	0.6206	.1689	.30	477
	July	.0008	0.5776	.0178	.23	511
	August	.0015	1.1374	.1361	.48	542
	September	.0010	0.9426	.0153	.29	482

**Figure 7:**
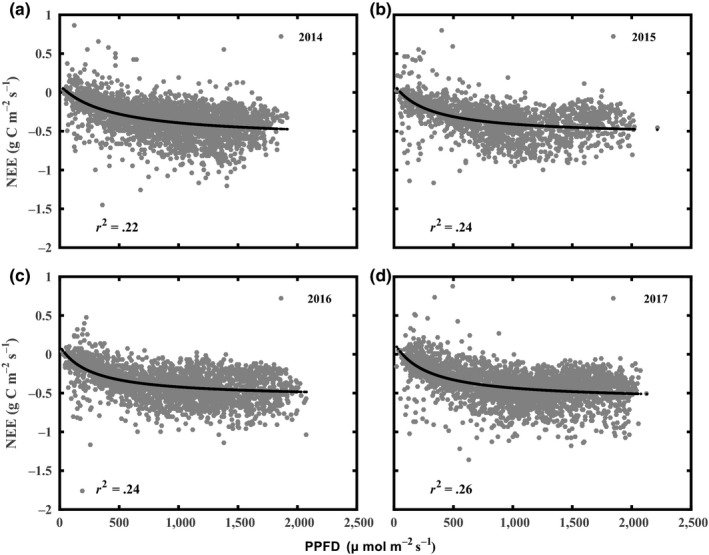
Response of ecosystem respiration (RE) to soil temperature (T_s_) data for the tea plantation, 2014–2017

A correlation analysis was used to investigate the effect of soil temperature on RE, and the result showed that nighttime RE increased exponentially with soil temperature during the four‐year study period. The soil temperature explained 39.55%–52.87% of the temporal dynamic of nighttime RE for the tea plantation in 2014–2017(Figure 7). The estimated temperature sensitivities of RE (Q10) were 1.67, 1.71, 1.41, and 1.48 in 2014–2017, respectively.

## 4 DISCUSSION

### 4.1 Seasonal dynamics in CO_2_ fluxes

The NEE and GPP of the tea plantation in this study exhibited distinct seasonal patterns that were similar to the dynamics of typical subtropical forests in Southeast China (Liu et al., 2006; Song et al., 2017; Yu et al., 2008), which are affected by climatic conditions and plant phenology (Baldocchi, 2008; Jans et al., 2010). The tea plantation was a moderate carbon sink throughout the study period except for the period after intense pruning interference during April each year (Figures 4 and 5). The suitable temperature and radiation conditions played a critical role in promoting the assimilation of CO_2_ in the tea plantation, and the GPP exceeded ER even in winter due to the evergreen tea plantation being able to assimilate CO_2_ in all seasons.

The annual NEE ranged from −301.51 to −182.40 g C m^−2 ^a^−1^ with an average value of −222.76 g C m^−2 ^a^−1^ over the four‐year study period; this magnitude of NEE is slightly higher than the average value of Asian and North American forests between 40°N and 60°*N* (−157 g C m^−2 ^a^−1^ and −180 g C m^−2 ^a^−1^, respectively) but is much lower than the NEE value of East Asian monsoon forests (−341 g C m^−2 ^a^−1^ from 20°N to 30°N and −368 g C m^−2 ^a^−1^ from 30°N to 40°N)(Yu et al., 2014). This NEE magnitude is also lower than the Moso bamboo forests in subtropical China (−602.7 g C m^−2 ^a^−1^)(Song et al., 2017). The daily maximum NEE values at our site (−4.45~−3.96 g C m^−2 ^day^−1^) were much lower than those from the paddy field ecosystem (−24.23 g C m^−2 ^day^−1^) in Kunshan Irrigation and Drainage Experiment Station, China (Yang, Xu, Liu, Zhang, & Wang, 2016), and lower than those from the subtropical hilly zone of mixed evergreen broadleaf and needle leaf forests (−7.4 g C m^−2 ^day^−1^) in Ningxiang County, Hunan Province, southern China (Jia et al., 2015). This observation is probably due to the intense pruning interference after tea harvest in April seriously affecting tea plantation growth, and the interference therefore decreased the plantation's carbon assimilation and resulted in lower carbon uptake capacity.

### 4.2 Effect of environmental drivers on CO_2_ fluxes

In this study, we found that in the studied subtropical hilly tea plantation, the seasonal variations of CO_2_ exchanges showed significant correlations with the seasonality of environmental variables, which indicated that the seasonal variations of CO_2_ exchange fluxes are affected by the seasonal variations of environmental factors. Therefore, understanding the underlying mechanisms between environmental drivers and CO_2_ exchange fluxes is important for estimating the CO_2_ budget in subtropical tea plantations. The most important environmental drivers of NEE were PPFD, T_a_, and P (Table 2). PPFD showed a significant influence on the daytime variations of NEE over the growing season in this tea plantation, and this relationship has been observed in other ecosystems (Niu et al., 2017; Song et al., 2017; Xie et al., 2015). NEE gradually increased with a higher PPFD during the growing season, and the PPFD explained 12%– 49% of the temporal dynamic in daytime NEE for the tea plantation (Figure 6). The NEE ceased to increase with PPFD when the PPFD went beyond approximately 1,000 μ mol m^−2^ s^−1^; this result was probably due to excessive light inhibition of the carbon uptake (Song et al., 2017; Turner et al., 2003; Ward, Evans, & Grimmond, 2013).

**Figure 8:**
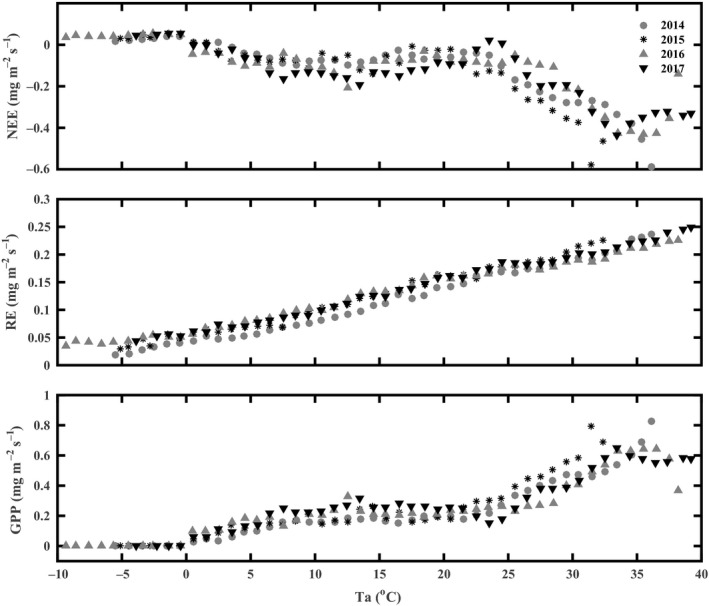
Response of NEE and its components GPP and RE to air temperature (T_a_) at the tea plantation, half‐hourly data during 2014–2017 were averaged by one degree air temperature interval

Many studies have reported that increases in temperature stimulate emissions of CO_2_; this observation is due to higher temperatures increasing enzyme activity and promoting microbial decomposition and plant respiration (Eze, Palmer, & Chapman, 2018; Loescher, Oberbauer, Gholz, & Clark, 2003; Niu et al., 2017; Yu et al., 2008). The responses of NEE and its components of GPP and RE to T_a_ were consistent with findings from previous studies (Garcia et al., 2017; Wagle et al., 2015). As the air temperature increased, NEE, GPP, and RE increased rapidly, but when the temperature went beyond 34°C, only RE still increased, as NEE and GPP appeared to decrease after approaching a maximum value at that temperature (Figure 8). In this study, GPP decreased faster than RE at higher temperatures, indicating that GPP was more sensitive to warming in this tea plantation. Future global warming may transform this subtropical tea plantation from a carbon sink to a carbon source.

### 4.3 Effect of management on CO_2_ fluxes

**Figure 9:**
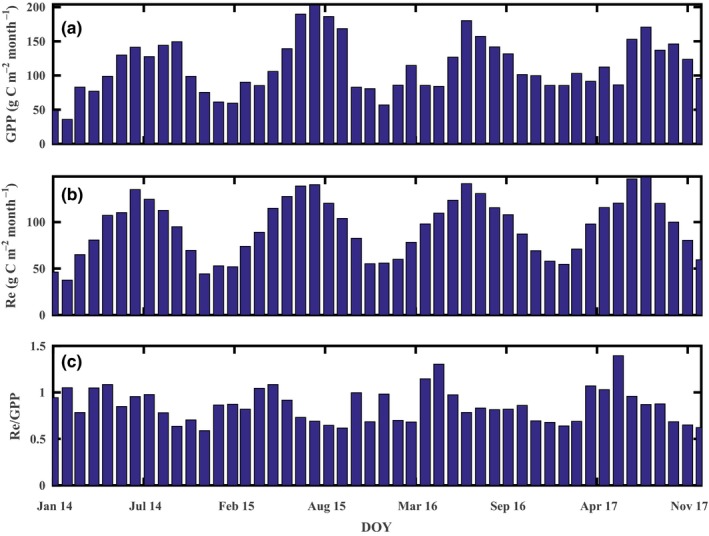
Monthly GPP, RE, and RE/GPP ratio at the tea plantation during the experimental periods (2014–2017)

We found that a monthly RE/GPP ratio of greater than 1 during April to June, which indicates a predominance of ecosystem respiration (Figure 9); this result is probably due to the intense pruning interference after tea harvest in April. Similar to cutting or grazing activities in grasslands (Hussain et al., 2011; Rutledge et al., 2015; Wohlfahrt et al., 2008), pruning leads to a sharp decrease in photosynthetic active matter, seriously affects tea plantation growth, and inhibits its CO_2_ assimilation. The pruned tea plant residues left in the field also provide a large substrate for microbial respiration, which might have led to the higher ER. These two combined effects led to the tea plantation to become a temporary carbon source during and after pruning. Pruning influenced the carbon dynamics by changing the aboveground biomass, plant residues, and soil temperature and moisture. Some studies found that permanent grazing could reduce the aboveground biomass and carbon uptake capacity in grasslands (Chen et al., 2011; Haferkamp & Macneil, 2004; Skinner, 2008), but other studies found that the higher photosynthetic efficiency of the regrowth of younger leaves will compensate for the carbon loss from grazing disturbance (Kjelgaard, Heilman, McInnes, Owens, & Kamps, 2008; Owensby, Ham, & Auen, 2006). Such pruning interference did not translate into this tea plantation being a carbon source at an annual time scale. The relatively warm and humid climate and phenology of the tea plantation allowed for year‐round growing conditions, as the ER/GPP ratio lower than 1 for the remainder of the year implies that this tea plantation had a strong carbon assimilation capacity between July and December (Figure 9).

Fertilizer addition is another management factor that affects the carbon dynamics in tea plantations, which will stimulate plant growth and microbial activity (Ammann, Flechard, Leifeld, Neftel, & Fuhrer, 2007; Schmitt, Bahn, Wohlfahrt, Tappeiner, & Cernusca, 2010; Skiba et al., 2013; Zeeman et al., 2010). Multiple studies reported that fertilizer additions will increase carbon uptakes (Eze et al., 2018; Moinet et al., 2016; Peichl, Leahy, & Kiely, 2011) or inhibit soil respiration and microbial respiration (Bowden, Davidson, Savage, Arabia, & Steudler, 2004; Janssens et al., 2010; Olsson, Linder, Giesler, & Hogberg, 2005; Ramirez, Craine, & Fierer, 2010), but other studies reported that the addition of N fertilizer results in a large carbon loss by the increase in soil microbial activity (Fang et al., 2012). Further studies are needed to improve our understanding of the effects of pruning and fertilization management activities and their complex interactions with environmental factors on carbon flux patterns.

## 5 CONCLUSIONS

The magnitude and temporal variations (diurnal, seasonal, and annual) of a carbon flux and its response to the environmental drivers of a tea plantation were quantified based on the eddy covariance technique. We found that the tea plantation was a net carbon sink on annual scales with an annual NEE that ranged from −182.40 to −301.51 g C/m^2^. PPFD explained the highest proportion of the variations in NEE and GPP (for NEE: *F* = 389.89, *p* < .01; for GPP: *F* = 1,018.04, *p* < .01), and the daily T_a_ explained the highest proportion of the variation in RE (*F* = 13,141.81, *p* < .01). The intense pruning interference after tea harvest during April resulted in a lower carbon uptake capacity. The results highlight the greater suppression of GPP than RE at higher air temperatures, which is important in predicting the effects of global warming on the carbon balance of this and other ecosystems.

## CONFLICT OF INTEREST

The authors declare that they have no conflict of interest.

## AUTHOR CONTRIBUTIONS

Hengpeng li conceived the study and designed the experiment; Jiaping Pang analyzed data and wrote the manuscript; Xuguang Tang and Jianwei Geng collected data and revised the manuscript.

## Data Availability

The carbon fluxes and meteorological data relevant to all analyses in this manuscript are available through Dryad (https://doi.org/10.5061/dryad.r9r0vj7).
